# KDM1A microenvironment, its oncogenic potential, and therapeutic significance

**DOI:** 10.1186/s13072-018-0203-3

**Published:** 2018-06-19

**Authors:** Tayaba Ismail, Hyun-Kyung Lee, Chowon Kim, Taejoon Kwon, Tae Joo Park, Hyun-Shik Lee

**Affiliations:** 1 KNU-Center for Nonlinear Dynamics, CMRI, School of Life Sciences, BK21 Plus KNU Creative BioResearch Group, College of Natural Sciences, Kyungpook National University, Daegu, 41566 South Korea; 2 School of Life Sciences, Ulsan National Institute of Science and Technology (UNIST), Ulsan, 44919 South Korea

**Keywords:** Histone demethylation, Carcinogenesis, Acute myeloid leukemia, KDM1A, TLL

## Abstract

The lysine-specific histone demethylase 1A (KDM1A) was the first demethylase to challenge the concept of the irreversible nature of methylation marks. KDM1A, containing a flavin adenine dinucleotide (FAD)-dependent amine oxidase domain, demethylates histone 3 lysine 4 and histone 3 lysine 9 (H3K4me1/2 and H3K9me1/2). It has emerged as an epigenetic developmental regulator and was shown to be involved in carcinogenesis. The functional diversity of KDM1A originates from its complex structure and interactions with transcription factors, promoters, enhancers, oncoproteins, and tumor-associated genes (tumor suppressors and activators). In this review, we discuss the microenvironment of KDM1A in cancer progression that enables this protein to activate or repress target gene expression, thus making it an important epigenetic modifier that regulates the growth and differentiation potential of cells. A detailed analysis of the mechanisms underlying the interactions between KDM1A and the associated complexes will help to improve our understanding of epigenetic regulation, which may enable the discovery of more effective anticancer drugs.

## Background

Epigenetic modifications are crucial for physiological development and steady-state gene expression in eukaryotes [1] and are required for various biological processes ranging from gene expression to disease pathogenesis [2]. DNA methylation, histone modifications, and post-translational modifications (PTMs) represent epigenetic alterations that may, alone or in combination, modify chromatin structure and gene activity by facilitating either gene activation or repression depending on the regulator type [3]. Histone methylation is the most versatile epigenetic modification involved in the establishment and maintenance of the epigenome [4]. The methylation of lysine residues at specific chromatin positions is essential for many processes, such as the activation and repression of transcription, transcriptional silencing mediated by heterochromatin, DNA repair, and inactivation of the X-chromosome, that are involved in the regulation of development. Additionally, these alterations may represent aberrant markers indicating the development of different types of cancer and other diseases [5–7].

**Fig. 1:**
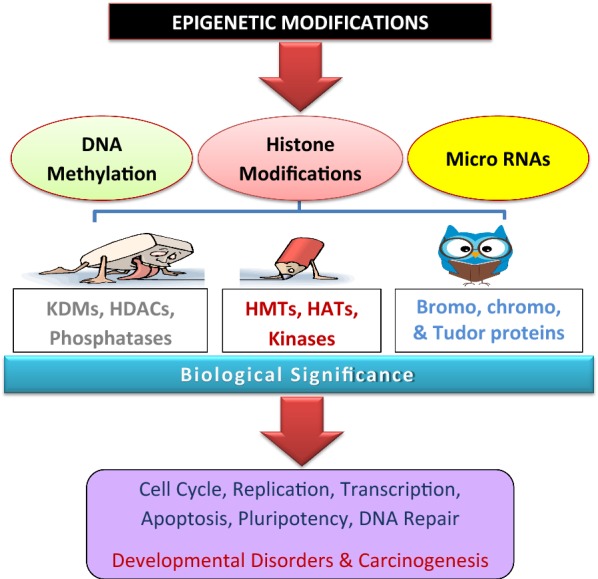
Epigenetic modifications and their biological roles. Epigenetic modifications are highly dynamic, and different types of modifications have been identified: DNA methylation, histone modifications, and microRNA-mediated modifications. Histone modifications are extremely versatile, and proteins known as “writers,” “readers,” and “erasers” are involved in this process. The writers, such as histone methyltransferases (HMTs), histone acetyltransferases (HATs), and kinases, add specific marks on sequences of amino acids on histone tails. Readers, such as proteins containing a bromo-domain, chromo-domain, or tudor-domain, are able to read these specific marks, which are further removed by the erasers, i.e., histone demethylases (KDMs), histone deacetylases (HDACs), and phosphatases. These histone modifiers, together with other epigenetic regulators, play an important role in the regulation of diverse biological functions [7]

Lysine residues can be mono-, di-, and tri-methylated in the nucleosome at strategic chromatin positions, and these methylated states have different functions [8]. Lysine no. 4, 9, 27, 36, and 79 of histone H3 and lysine 20 of histone H4 are the most frequently studied histone methylation sites and are associated with various biologically significant processes [9]. These methylation marks were considered stable and irreversible prior to the discovery of the molecules termed “erasers,” i.e., histone demethylases [10]. Shi et al. made the first discovery of histone lysine demethylase in 2004 [11], and this led to the establishment of new paradigms in the field of epigenetics (Fig. 1). These epigenetic regulators have been clustered into two subclasses [12]: one, including the majority of these regulators, containing a jumonji domain that depend on iron and oxoglutarate as cofactors [13], and the other comprising of two lysine-specific demethylases that contain an amine oxidase domain and rely on flavin adenine dinucleotide (FAD) as their cofactor [14]. All histone modifiers were shown to have important roles in gene regulation and epigenome establishment [15]. However, lysine-specific histone demethylase 1A (KDM1A/LSD1), being the first identified histone demethylase, has been widely explored, and numerous studies have described its biological roles [16]. KDM1A represents an important enzyme that plays significant roles in the regulation of embryonic development and differentiation [17]. Furthermore, together with associated proteins, this protein regulates many physiological processes involved in the shape and identity determination of stem and progenitor cells and also plays a role in their differentiation into specialized cells, i.e., hematopoietic, neural, mesenchymal, sperm, and fat cells [18, 19]. KDM1A has also been associated with the development of a variety of pathological conditions, such as cancer, neuronal disorders, and viral infections [20].

The functional diversity of KDM1A is supported by its complex structure [19]. In this review, we focus on the microenvironment of KDM1A in carcinogenesis and its structure, which is involved in the maintenance of its microenvironment by establishing complex interactions with a variety of transcriptional factors, promoters, activators, corepressors, and noncoding RNAs. Additionally, we discuss the versatile nature of KDM1A as an epigenetic modifier, regulating the expression of a number of genes involved in epithelial–mesenchymal transition (EMT). Moreover, the potential and challenges associated with KDM1A therapeutic targeting are summarized here, together with a brief description of the similarities and differences between this demethylase and its recently discovered homolog, KDM1B, the other member of the FAD-dependent demethylase family.

### Structural analysis of KDM1A

**Fig. 2:**
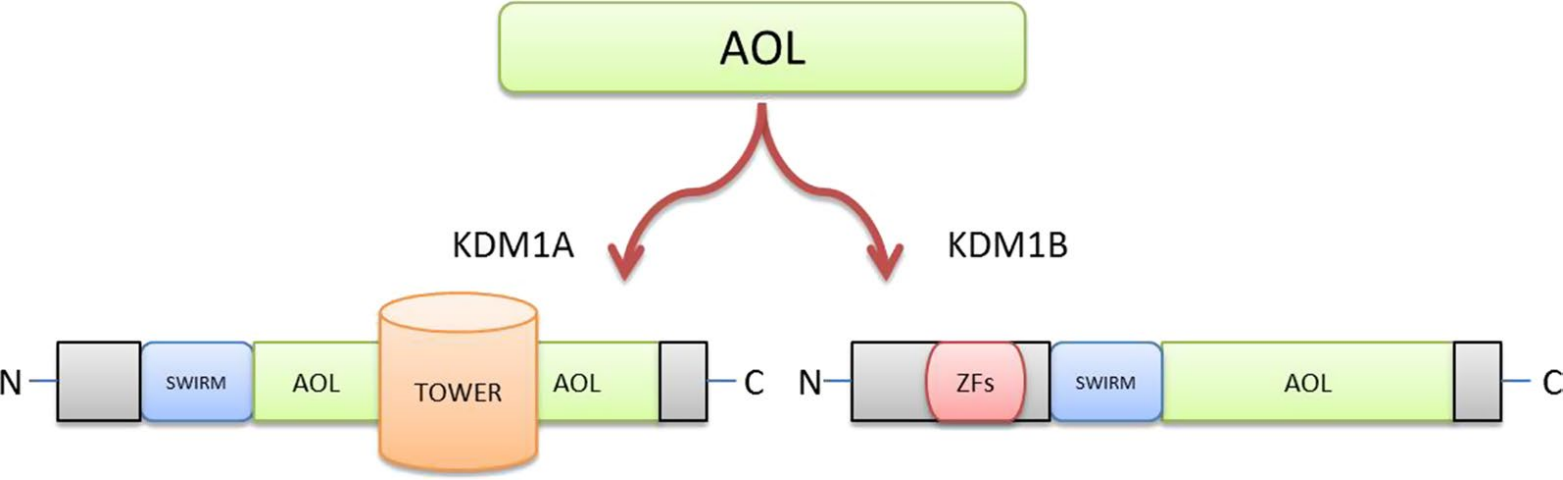
Structural domain analysis of KDM1A and KDM1B. Lysine-specific demethylase 1A and 1B each contain an amine oxidase-like domain and a SWIRM (SWI3, RSC8, and Moira) domain. The SWIRM domain of KDM1A is specific for chromatin–protein interactions, while that of KDM1B interacts with glyoxylate reductase 1 (GLYR1). KDM1A and 1B are characterized by TOWER and zinc finger (ZF) domains. The tower domain of KDM1A divides the AOL domain into two halves and is involved in interactions with different protein complexes [18]

KDM1A, the first demethylase to be identified, is also known as LSD1, AOF2, BHC110, or KIAA0601 [21], and structural analyses have demonstrated that this protein contains an amine oxidase-like domain (AOL) [22]. Initially, KDM1A was considered a nuclear protein, similar to the FAD-dependent amine oxidases, but it was later shown to be a demethylase [23]. Despite the structural similarity between the AOL domain of KDM1A and the amine oxidase domains of other amine oxidases, it exhibits numerous differences, e.g., it contains a SWIRM (swi3p/Rsc8p/Moira) domain at its N-terminus, which plays a significant role in protein–protein interactions [24]. Furthermore, KDM1A contains a TOWER domain (90-residue insert), dividing the AOL domain into two subdomains (Fig. 2) [25, 26]. One subdomain of AOL interacts with the SWIRM domain, forming a core structure that binds FAD, while the other specifically binds the substrate [27]. The FAD-binding subdomain of AOL is similar to the amine oxidase domain of other amine oxidases, but the substrate-binding subdomain contains a large binding pocket with acidic features at its surface to facilitate the accommodation of long basic histone tails by maintaining specific interactions with the first 20 amino acids of histone 3 (H3) [28]. Moreover, the active site of KDM1A possesses side chains at its rim that are negatively charged in order to establish interactions with the tail of the histone substrate through hydrogen bonding and salt bridges [29]. This unique KDM1A binding site mediates its demethylation function and enables KDM1A to recognize a wide range of nonhistone substrates [30–32].

The SWIRM domain of KDM1A does not bind with DNA molecules, as it is specific for protein–protein interactions and maintains the structural integrity of protein substrates [24, 27]. Furthermore, it is involved in altering the substrate specificity of KDM1A from H3K4 to H3K9 [33, 34]. In addition to the different structural domains of KDM1A, its demethylation capacity depends on the number of residues in the substrate-binding site and at the interface of the AOL-SWIRM domain [35, 36]. Some of these residues affect the catalytic capacity of KDM1A, whereas others affect protein structure and substrate interactions [37]. KDM1A shows high substrate specificity, and mutations in substrates hinder the physical enzyme–substrate interactions, ultimately inhibiting target demethylation [31, 38]. Therefore, the demethylation capacity of KDM1A is affected by residual PTMs.

KDM1A can recognize p53, E2F1, and DNMT1, in addition to H3 [32, 39, 40], although no structural homology exists between histones and these nonhistone substrates [41]. It is possible that the unique structure of KDM1A and its microenvironment allows interactions with a wide range of substrates.

### Comparison between KDM1A and KDM1B

KDM1B (also known as LSD2 or AOF1) represents the second FAD-dependent AOL-domain-containing demethylase belonging to the LSD family of histone demethylases [21]. KDM1A and KDM1B share many structural properties, such as the presence of the catalytic AOL domain and SWIRM domain specific for chromatin and protein interactions. Unlike the SWIRM domain of KDM1A, the SWIRM domain of KDM1B is closely associated with the AOL domain and is involved in maintaining interactions with glyoxylate reductase 1 (GLYR1), a positive regulator of demethylation [42]. A coiled loop, unique to KDM1B and absent from the SWIRM domain of KDM1A, is involved in the establishment of this interaction [26].

Although KDM1A and KDM1B both contain the AOL and SWIRM domains, but the structure of these domains vary between these two homologs. KDM1A and KDM1B interact with different proteins and exhibit essentially different genomic profiles. The primary difference between these LSD family members is that KDM1A is involved in the formation of the RE1-silencing transcription factor (REST) corepressor (CoREST) complex through the TOWER domain that is absent in KDM1B [29, 35, 37]. Instead, KDM1B possesses a zinc finger domain at its amino terminus (Fig. 2) that is unique to KDM1B and is composed of two individual zinc fingers, i.e., an N-terminal C_4_H_2_C_2_-type zinc finger and a CW-type zinc finger [34, 43]. CW-type zinc finger domains are found in many chromatin remodeling protein complexes and have the ability to bind to methylated histone proteins [44–46]. In contrast to other CW-type zinc finger domains, however, the CW-type zinc finger domain of KDM1B does not bind to the methylated H3 tail [43]. This N-terminal zinc finger domain is also required for the binding of FAD cofactor [43]. Moreover, in addition to its roles in protein–protein and DNA–protein interactions, the N-terminal zinc finger domain functions as a structural scaffold via intramolecular interactions [43]. While it is clear that the unique amino terminal zinc finger domain and SWIRM domain of KDM1B are crucial for the demethylase activity of this enzyme, their detailed mechanisms of action are currently unknown [43]. From a functional point of view, KDM1B differs from KDM1A in its ability to demethylate both core histones and nucleosomal substrates [26].

### Interacting partners of KDM1A, its substrate specificity, and functional diversity

**Fig. 3:**
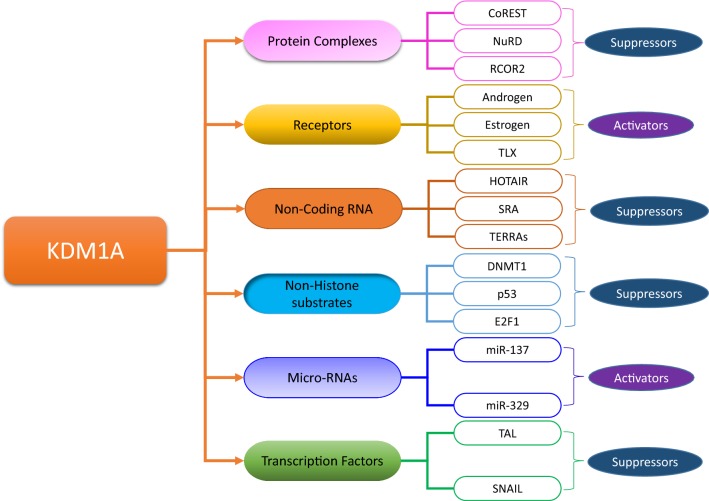
KDM1A interacting partners and functional diversity. The microenvironment of KDM1A contains various protein complexes (e.g., CoREST, NuRD, and RCOR2), receptors (estrogen, androgen, and TLX), noncoding RNAs (HOTAIR, SRA and TERRAs), microRNAs (miR-137 and miR-329), nonhistone proteins (p53, E2F1, and DNMT1) and transcription factors (TLA and SNAIL). The interaction of KDM1A with these diverse factors allows the dynamic regulation of different biological processes through the suppression and the activation of target gene expression depending upon the type of its interacting partner, i.e., the interaction of KDM1A with miR-137 downregulate the expression of KDM1A and in turn led to the differentiation of cells by activating the associated genes while its association with CoREST results in downregulation/suppression of target genes

KDM1A was initially identified as a binding partner of CoREST [37, 47]. KDM1A, together with CoREST, is frequently found in many other larger protein complexes, in which it acts as a scaffold by joining the deacetylase and demethylase activities into a single complex [31, 38, 48–50]. The association of KDM1A with the CoREST complex allows it to demethylate the nucleosome [51]. In addition to CoREST, its paralogs, i.e., CoREST2 and CoREST3, also bind to KDM1A and regulate the functional activities of this demethylase upon incorporation into larger protein complexes [52, 53]. However, CoREST2 exhibits a decreased ability to facilitate KDM1A-mediated nucleosome demethylation [52]. Unlike CoREST2, competitive inhibition of KDM1A-mediated nucleosomal demethylation is observed for CoREST3; thus, it exhibits even stronger antagonistic behavior [53]. The functional diversity of KDM1A depends on its interacting partners (Fig. 3), including protein complexes, transcription factors, receptors, noncoding RNAs, and nonhistone proteins [31, 38, 54].

KDM1A interacts with orphan nuclear hormone receptor TLX and plays a role in the regulation of neuronal cell differentiation [55]. TLX recruits the CoREST/KDM1A/HDAC1 complex in KDM1A-dependent manner via direct interactions with the AOL and SWIRM domains of KDM1A to facilitate H3K4 demethylation and H3 deacetylation and to maintain other downstream genes in a repressed state [56]. The TLX/KDM1A complex also regulates neuronal stem cell proliferation [57]. The interaction of KDM1A with TLX aids in the timely regulation of neuronal proliferation and differentiation events [58].

The transcription factor TAL1 is involved in the regulation of the normal processes of hematopoiesis and leukemogenesis and functions as an activator and repressor of transcription [59]. These transcriptional repression and activation activities of TAL1 are maintained by its interactions with a variety of complexes and depend upon many other factors [60–64]. TAL1 acts as the binding partner of KDM1A in association with the CoREST/HDAC complex and functions as a repressor of erythroid-specific genes in progenitor cells prior to differentiation events [65]. During the early stages of differentiation, the interaction of KDM1A and TAL1 is lost, and the repression of these erythroid-specific genes is eliminated. The PTM of TAL1 plays a role in its binding to KDM1A [66]. The phosphorylation of TAL1 leads to the dissociation of the KDM1A complex from TAL1 and mediates transcriptional activation [65, 66].

KDM1A also interacts with C-terminal binding proteins (CtBP), which are well-known repressors of mammalian gene expression [67]. The interaction of KDM1A with CtBP was known before the discovery of its demethylase activity [50] and is implicated in a variety of CtBP functions, such as the regulation of pituitary gland development [68], repression of the tumor-suppressor gene *BRCA1* [69], and activation of tissue-specific genes in endocrine cells in the gastrointestinal tract [70, 71]. However, the more established role of the KDM1A and CtBP association is the suppression of E-cadherins, proteins involved in the process of EMT [50, 72, 73].

The interaction of KDM1A with the nucleosome remodeling and histone deacetylase (NuRD) complex implicates KDM1A in a variety of biological processes [74, 75], since NuRD regulates various biologically significant events, ranging from development to the progression of different types of malignancies [76]. By binding with the NuRD complex, KDM1A catalyzes the demethylation of nucleosome substrates [75]. Instead of the CoREST complex, MTA proteins that structurally resemble CoREST recruit KDM1A and mediate the demethylation reaction of KDM1A [77]. KDM1A, in association with the NuRD complex, is involved in the repression of the TGF-β signaling pathway and the inhibition of EMT [75].

**Fig. 4:**
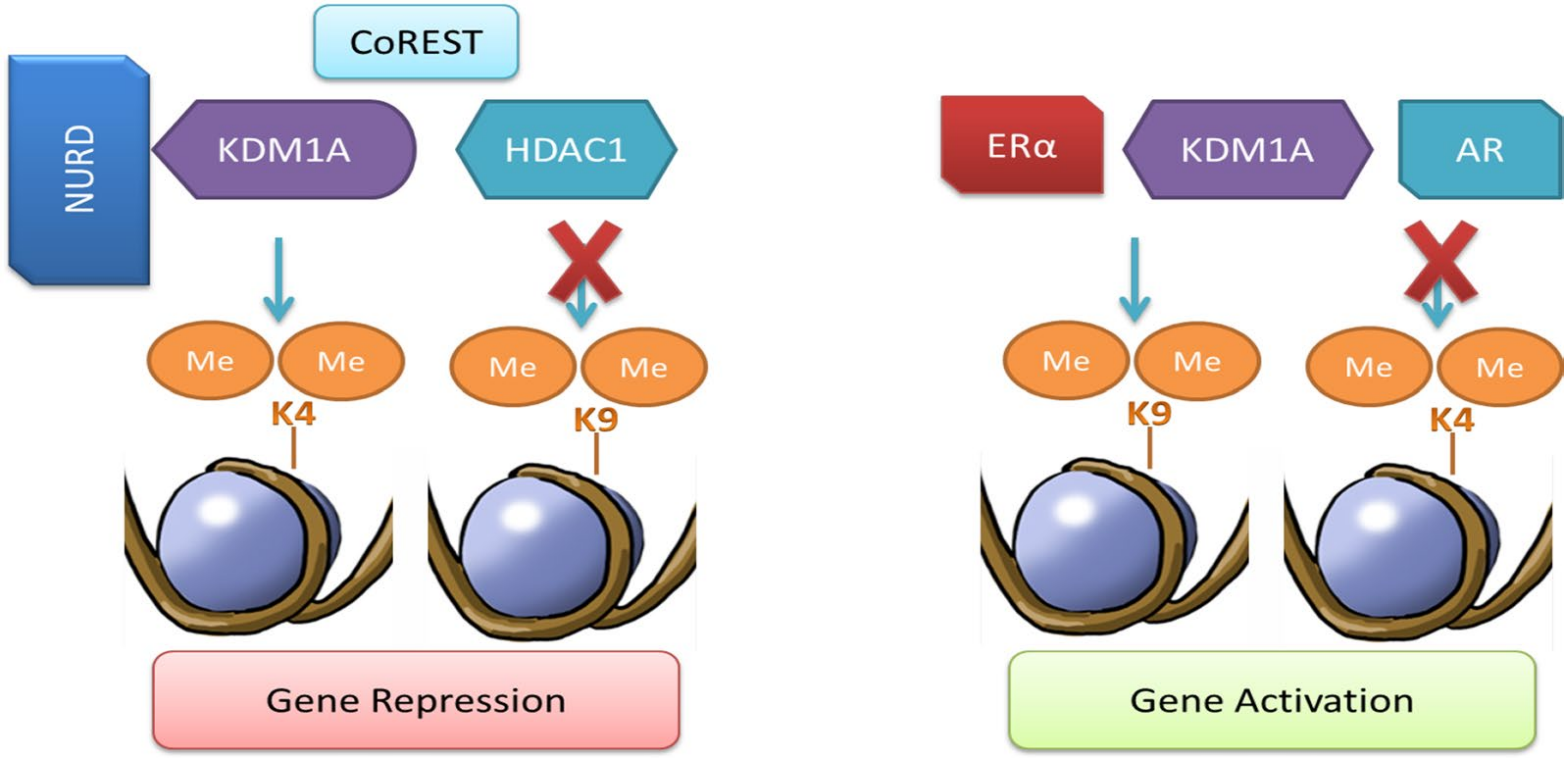
Substrate specificity and regulation of gene expression by KDM1A. The binding of KDM1A to the CoREST and NuRD complex allows the demethylation of H3K4me1/2 and leads to the inhibition of target gene expression, but this complex cannot catalyze the demethylation of the lysine 9 of histone 3 (H3K9me1/2). The interaction between KDM1A and the androgen and estrogen receptors alters its substrate specificity from H3K4me1/2 to H3K9me1/2, allowing for the regulation of target gene expression [18]

In addition to the aforementioned interactions of KDM1A, it also takes part in nuclear hormonal signaling by interacting with androgen receptors (ARs) [78] and estrogen receptors (ERs). ARs are associated with the regulation of prostate function, from normal tissue development to the initiation and progression of metastasis [79]. KDM1A, in association with ARs, changes its substrate specificity from H3K4me2 to H3K9me1/2 (Fig. 4) [78]. This change facilitates the activation of AR-mediated gene transcription [78]. Protein kinase Cβ1 (PKCβ1) plays a role in the substrate switching of the KDM1A/AR complex from H3K4 to H3K9 at target genes by phosphorylating H3T6 [80]. AR target genes can also be repressed by KDM1A as, unlike ARs, KDM1A resides at the promoters of AR target genes, even in the absence of androgen treatment, and at that time, these genes are in a repressed state [78, 81]. Moreover, a negative feedback loop is formed by KDM1A/AR under high androgen levels [82]. In this state, KDM1A is recruited at the enhancers of target genes by AR and facilitates target gene repression by demethylating H3K4 [82].

KDM1A also interacts with estrogen receptor alpha (ERα), which is associated with estrogen signaling in estrogen-responsive tissues, and any impairment in its function can lead to the genesis and progression of various types of cancers [83, 84]. KDM1A functions as both an activator and repressor of genes in association with ERα, similar to the mechanism by which KDM1A associates with ARs [85].

Because of the interaction of KDM1A with a wide variety of complexes, it has been suggested that the microenvironment of KDM1A dictates its substrate specificity and leads to the growing functional complexity of this FAD-binding demethylase.

### KDM1A–RNA interactions

KDM1A regulates the expression of target genes through histone demethylation. In addition to other molecules, KDM1A interacts with several RNAs, including microRNAs such as miR-137 [25]. miR-137 is expressed in the nervous system and is significant for regulation of neural stem cell differentiation [58]. It regulates the expression of KDM1A by targeting its 3′ untranslated-region (UTR), leading to the differentiation of neural embryonic stem cells [58]. KDM1A is also involved in the fate determination of neural stem cells by acting as the TLX corepressor (nuclear receptor subfamily 2 group E member 1) that targets miR-137 and inhibits its expression [58]. These molecules form a regulatory loop that controls the differentiation of neural stem cells. Recently, miR-329 was also shown to target the 3′-UTR of KDM1A, suppressing its expression [86]. Notably, in addition to interactions between KDM1A and microRNAs, an association between KDM1B and the microRNA miR-215 has also been observed [87]. The post-transcriptional induction of miR-215 through the HIF-Drosha complex inversely correlates with KDM1B expression and plays a role in the adaptation of glioma-initiating cells (GICs) to hypoxic conditions [87].

In addition to microRNAs, KDM1A interacts with long noncoding RNAs (lncRNAs) [88]. lncRNAs have been implicated in several types of cancers, and they function as regulators of gene transcription by acting as scaffolds for chromatin-modifying complexes [89–91]. The overexpression of the lncRNA HOTAIR has been observed in many types of cancer [92]. This noncoding RNA interacts with the KDM1A/CoREST complex, mediates its interaction with the polycomb repressive complex 2 (PRC2), and assists in its recruitment to the *HOXD* locus [88] to downregulate the expression of tumor-suppressor genes [93]. Furthermore, the HOTAIR-mediated KDM1A/PRC2 complex positively regulates the transcription factor NFAT5, which is involved in angiogenesis and the progression of breast cancer [94]. KDM1A has also been found to interact with another breast cancer-associated lncRNA, steroid receptor RNA activator (SRA) [95]. However, in this case, the interaction is mediated by progesterone receptors (PRs) [96]. KDM1A was also shown to interact with TERRAs (RNAs encoded by telomeric sequences) and plays a role in the DNA damage of uncapped telomeres [97].

### EMT and the KDM1A microenvironment

**Fig. 5:**
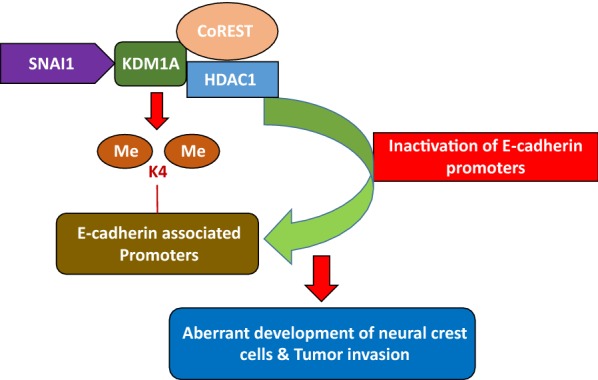
Role of KDM1A in epithelial–mesenchymal transition. SNAI1 recruits the KDM1A corepressor complex, leading to the demethylation of H3K4me2 in the histone tails of E-cadherin-associated promoters. This demethylation ultimately inactivates these E-cadherin associated promoters. This SNAI1-mediated interaction of KDM1A with E-cadherin controls the processes of neural development and tumorigenesis

EMT is crucial for embryonic development and tumor metastasis and is characterized by the alteration/reprogramming of epithelial cells [98], which acquire migratory properties and are transformed into mesenchymal cells [99]. EMT is a complex process regulated by a number of factors and signaling pathways and is crucial for the development of the neural crest and mesoderm formation [100]; it also plays important roles in carcinogenesis and tumor propagation [100]. KDM1A is involved in EMT through interactions with the members of the SNAI1 family of zinc finger transcription factors, including SNAI1 (SNAIL) and SNAI2 (SLUG) [101, 102]. The expression of SNAI1 and E-cadherin is a hallmark of carcinoma development and metastasis. The downregulation of E-cadherin or both of these proteins occurs following the interaction of SNAI1 with KDM1A. SNAI1 recruits the KDM1A corepressor complex through its SNAG domain, leading to the demethylation of H3K4me2 in the histone tail of E-cadherin-associated active promoters [101]. The inactivation of E-cadherin promoters drives the aberrant development of neural crest cells and increases tumor invasion and propagation (Fig. 5). The interactions between KDM1A and SNAI1, followed by interactions with E-cadherin, enable KDM1A-mediated control of carcinogenesis [101].

### KDM1A microenvironment in oncogenesis

The complex structure of KDM1A allows it to interact with a wide variety of protein complexes, transcription factors, hormone receptors, and different types of RNAs. Its diverse microenvironment has been implicated in the genesis and progression of cancer (Table 1) [20, 103, 104]. KDM1A has been studied for its roles in several malignancies, which are described below. While investigations into the role of KDM1B in oncogenesis are lacking, the enhanced expression of KDM1B has been observed in breast cancer, and its targeted repression is observed in glioblastoma [87, 105].

**Table 1:** Roles of KDM1A in oncogenesis

Type of cancer	KDM1A interactions	Functional role of KDM1A	References
Prostate cancer	Androgen receptor	↓E-cadherin, ↑VEGF-A	[104, 105, 107]
Breast cancer	Estrogen receptor (ERα), CAC2, β-catenin	↓p57Kip2, ↓LEFTY1, ↓BRCA1	[119, 120, 122–124]
Oral cancer	E2F1 signaling pathway	↑Cell proliferation	[130]
Colorectal cancer	Wnt/β-catenin pathway	↓DKK1, ↑LGR5	[134, 135]
Neuroblastoma	miR-137	↑Poorly differentiated cells	[111, 112]
Glioblastoma	GSK3β	↓BMP2, ↓CDKN1A, ↓GATA6	[107, 114]
Acute myeloid leukemia	MLL-AF9	↑Activation of oncogenes	[145]
T-cell acute lymphoid leukemia	Notch signaling pathway, TAL1/SCL	↑Activation and ↓repression of Notch target genes	[61, 62, 150]

KDM1A interact with different signaling pathways and interacting partners. This interaction enables KDM1A to play role in development of different types of cancers. ↓ indicates the down regulation while ↑ denotes the upregulated expression of different genes and factors

#### Prostate cancer

Prostate cancer is one of the most common cancers in males, and the overexpression of KDM1A plays an important role in prostate cancer initiation and progression [106]. Although there are studies that describe very little to no overexpression of KDM1A in prostate cancer cell lines [107, 108], the overexpression of KDM1A has been found to be associated with prostate cancer progression and recurrence [109]. In prostate cancer, the substrate specificity of KDM1A changes from H3K4me1/2 to H3K9me1/2 through its interactions with ARs [110]. The increased expression of KDM1A, accompanied by a reduction in E-cadherin expression, can be used as a predictive marker for prostate cancer progression and metastasis [111]. KDM1A regulates the expression of vascular endothelial growth factor A (VEGF-A), which is important in prostate cancer progression [109, 112]. The enhanced expression of VEGF-A was shown to be correlated with KDM1A overexpression [113]. Moreover, the increase in KDM1A expression is responsible for the androgen-independent transition of prostate cancer cells (LNCaP) [114], protecting them from apoptosis by activating AR signaling and inhibiting p53 signaling [4, 115]. The inhibition of KDM1A leads to the suppression of gene transcription facilitated by ARs and the inhibition of prostate cancer progression [78].

#### Neuroblastoma

The most common extracranial solid tumor of childhood (neuroblastoma) is associated with aberrant overexpression of KDM1A [116]. High expression of KDM1A was observed in poorly differentiated neuroblastoma cells, and downregulation of KDM1A was found in differentiated neuroblastoma cells [117]. However, more detailed investigations are required to understand the interaction between KDM1A and genes associated with neuroblastoma. It has been found that the microRNA miR-137 acts to suppress KDM1A expression in neuroblastoma. miR-137 is expressed at low levels in aggressive neuroblastoma tumors but directly targets KDM1A [118]. Thus, increasing the expression of miR-137 in neuroblastoma cells may serve as an effective therapeutic strategy for the treatment of aggressive neuroblastoma [118].

The overexpression of KDM1A has also been observed in a tumor closely related to neuroblastoma, i.e., medulloblastoma [119]. Medulloblastoma is the leading cause of death among childhood malignancies, and currently available treatments for this tumor are associated with certain neurological disabilities among survivors [119]. The targeting of KDM1A in this tumor may lay the foundation for effective medulloblastoma therapy [119].

#### Glioblastoma

The phosphorylation of KDM1A has been observed during the progression of human glioblastoma. Glycogen synthase kinase 3β (GSK3β) promotes the de-ubiquitination of KDM1A by phosphorylation. Ubiquitin-specific peptidase 22 (USP22) then recognizes phosphorylated KDM1A and stabilizes it by de-ubiquitination [113]. An increase in the expression of GSK3β- and USP22-dependent KDM1A leads to the demethylation of H3K4, which further promotes the transcriptional repression of bone morphogenetic protein 2 (BMP2), cyclin-dependent kinase inhibitor 1A (CDKN1A), and GATA-binding protein 6 (GATA6). KDM1A-mediated transcriptional repression of these genes underlies the self-renewal of cancer stem cells and glioblastoma progression [113, 120].

The targeted suppression of KDM1B by miR-215 has been observed in glioblastoma initiating cells (GICs) that are essential for glioblastoma occurrence and re-occurrence [87]. miR-215 is post-transcriptionally induced by hypoxia-inducible factor (HIF) via interactions with the HIF-Drosha complex [87]. The enhanced expression of miR-215 is negatively correlated with KDM1B expression and positively correlated with HIF1α expression in glioblastoma progression [87].

#### Breast cancer

Breast cancer is among the most common malignancies associated with an increased mortality rate in women [121, 122]. The formation and progression of breast cancer is influenced by different genetic and epigenetic abnormalities [123]. The overexpression of KDM1A can be considered an early event in breast cancer tumorigenesis [124]. H3K4 demethylation by KDM1A affects the expression of the *p57Kip2* gene, which encodes a cyclin-dependent kinase inhibitor that is essential for breast tumor development [125]. KDM1A expression is also required for the proper functioning of ERα, which is highly expressed in the majority of breast tumors [126]. The recruitment of estrogen-bound ERα to estrogen-responsive gene promoters is attenuated by the inhibition of KDM1A, and this exerts anti-proliferative effects in breast cancer [127]. Moreover, CDK2-associated cullin (CAC2) interacts with KDM1A and decreases the function of ERα co-activator [128]. Additionally, KDM1A interacts with β-catenin and regulates the expression of the tumor-suppressor gene *LEFTY1* [129]. The mRNA levels of KDM1A and β-catenin are inversely correlated with the expression of *LEFTY1*.

KDM1A overexpression has been observed in ER^−^ breast cancers as well and was shown to correlate with a reduction in *BRCA1* (a familial susceptibility gene for breast cancer) expression [130]. The dysregulation of BRCA1 expression induces a basal-like phenotype in breast cancer cells. Ubiquitin-specific peptidase 28 (USP28) plays a role in the stabilization of KDM1A in multiple cancers, including breast cancer, through its de-ubiquitination [131]. The phosphorylation of KDM1A at Ser112 is required for breast cancer metastasis, as the phosphorylated protein inhibits E-cadherin expression [132]. The increased expression of histone-modifying enzymes, such as KDM1A, histone deacetylase 2 (HDAC2), and NAD-dependent deacetylase sirtuin-1 (SIRT1), was observed in breast cancer samples, and their overexpression was shown to be associated with reduced survival and a shorter period of tumor relapse [133]. Furthermore, the expression levels of KDM1A and HDAC isozymes are correlated, i.e., *KDM1A* knockdown induces a decrease in the expression of HDAC5 in triple-negative breast cancer [134], while the depletion of HDAC5 leads to the accumulation of H3K4me2 [134]. This suggests that KDM1A and HDAC may represent potential prognostic factors for breast carcinogenesis.

In addition to KDM1A, its homolog KDM1B is highly expressed in breast cancer, particularly in invasive tumors [105]. The enhanced expression of KDM1B in MDA-MB-231 cells has been shown to alter the expression of key epigenetic regulators, i.e., KDM1A, HDAC1/2, and DNMT3B; stimulate cellular proliferation; and enhance colony formation in soft agar while decreasing motility and invasion [135]. Additionally, KDM1B overexpression in MDA-MB-231 cells led to increased tumor growth, facilitated mammosphere formation, and resulted in the induction of pluripotent stem cell markers, i.e., NANOG and SOX2. Thus, KDM1B also plays significant and multifaceted roles in breast cancer progression and the enrichment of cancer stem cells [135]. Knockout of *KDM1B* increases the expression of many key silenced genes that are significant in breast cancer development [105]. However, a detailed investigation of the underlying mechanism of KDM1B in breast cancer metastasis is needed.

#### Oral cancer

Oral cancer is the most common cancer among developing countries, and KDM1A expression is upregulated in oral tumors compared to levels in normal oral tissues [136]. KDM1A regulates the E2F1 signaling pathway in oral cancer and increases cell proliferation [137]. Moreover, the inhibition of KDM1A alleviates E2F1 signaling activities, and its overexpression leads to poor clinical outcomes [137]. KDM1A serves as a novel biomarker and early prognostic factor for oral and tongue cancer [138].

#### Colorectal cancer

The enhanced expression of KDM1A is also observed in colon and colorectal tumors [139, 140]. KDM1A plays a role in activating the Wnt/β-catenin signaling pathway, but, at the same time, downregulates the signaling pathway antagonistic to the colorectal cancer-related gene dickkopf-1 (*DKK1*) [141]. Moreover, increased expression of KDM1A is also associated with the expression of leucine-rich repeat-containing G-protein-coupled receptor 5 (LGR5), a well-known colorectal cancer stem cell marker [142]. The inhibition of KDM1A attenuates Wnt/β-catenin signaling and diminishes colorectal cancer progression by downregulating the expression of LGR5 [142].

The expression of KDM1A is also associated with reduced expression of CDH1, which results in colon cancer metastasis [139]. Moreover, the upregulated expression of KDM1A significantly reduced the expression of E-cadherin in samples of advanced colon cancer and distant metastases [139].

#### KDM1A in other malignancies and sarcomas

The enhanced expression of KDM1A has also been observed in pancreatic cancer [117], non-small-cell lung carcinoma [143, 144], and human epithelial ovarian cancer [145]. Furthermore, KDM1A was shown to be involved in bladder cancer [146], while the immunoreactivity of KDM1A was shown to be elevated in hepatocellular carcinoma [147]. The upregulation of KDM1A is also observed in chondrosarcoma, Ewing’s sarcoma, and osteosarcoma [148]. Moreover, a US Food and Drug Administration-approved drug that inhibits KDM1A was also found to inhibit chondrosarcoma, Ewing’s sarcoma, osteosarcoma, and rhabdomyosarcoma cell growth in vitro [148]. These results demonstrate that KDM1A represents an important epigenetic regulator that is essential for cell growth and differentiation due to its interactions with various factors. These KDM1A-induced alterations in gene expression levels are associated with cellular oncogenic potential.

### KDM1A in acute myeloid leukemia (AML)

Hematopoiesis is a complex process regulated by various epigenetic modifiers [149]. During physiological hematopoiesis, alterations in gene expression in stem cells are responsible for the differentiation of mature blood cell lineages and removal of the stem cell identity [66]. In AML, hematopoietic stem cell control is disturbed, and these stem cells develop in an unlimited manner, exhibiting self-renewal, increased proliferation, and poor differentiation [150]. KDM1A and the mixed-lineage leukemia gene (*MLL*) play a role in cell differentiation during hematopoiesis [151].

Experimental mouse and human studies of MLL-AF9 leukemia have demonstrated that *KDM1A*-knockout cells differentiate efficiently and do not form colonies [152]. The accumulation of H3K4me2 at the promoter region of *MLL*-*AF9* was observed in the absence of KDM1A [153]. The expression of KDM1A was shown to be associated with the activation of oncogenes specific for leukemia stem cells [151, 154]. Moreover, KDM1A is an effective drug target for AML therapy [153]. A number of KDM1A inhibitors have been investigated for their potential to inhibit growth in AML by inducing KDM1A inhibition [155]. However, single-agent therapy is not suitable for AML because it is associated with an increased risk of remission [156, 157]. Hence, combinatorial approaches including HDAC inhibitors are under investigation for curative treatment of AML [158]. In addition, the activation of oncogenic target gene programs and the recruitment of various protein complexes by KDM1A should be further studied.

### KDM1A in T cell acute lymphoid leukemia (T-ALL)

KDM1A overexpression has been observed in T-ALL, in which it was shown to be characterized by aberrant Notch signaling and T-cell progenitor malignancy [66], originating from mutations in the *NOTCH1* gene. KDM1A is a part of the multifunctional Notch complex, acting as a NOTCH1 target gene modifier [159]. KDM1A-mediated gene activation and repression has been observed in T-ALL [160]. The activation of NOTCH1 target genes by DNA-binding complex CSL occurs in the presence of NOTCH1, whereby KDM1A preferentially targets H3K9me2, while in the absence of NOTCH1, KDM1A demethylates H3K4me2 residues, leading to the suppression of NOTCH1 target gene expression [161]. Therefore, KDM1A acts as a mechanistic switch for the activation and repression of NOTCH1 target genes. The inhibition of KDM1A is associated with growth arrest and alterations in T-ALL, similar to the effects of NOTCH1 silencing [160].

The association of KDM1A with the hematopoietic stem cell transcription factor TAL1/SCL was shown to be important in the differentiation of stem cells, while its deregulation was associated with T-ALL development [65]. The phosphorylation of TAL1 at Ser172 by protein kinase A (PKA) induces the dissociation of KDM1A/TAL1, consequently activating target genes by inducing the expression of H3K4me2 in promoter regions [66].

### KDM1A as a therapeutic target and associated challenges

The identification of functional significance of KDM1A in various malignancies and developmental disorders shows that this demethylase may represent a potent therapeutic target. The development of an efficient KDM1A inhibitor is in progress [162]. The structural similarity between monoamine oxidases (MAOs) and KDM1A has led to the investigation of anti-MAO compounds as inhibitors of KDM1A. Tranylcypromine, an MAO inhibitor, can inhibit KDM1A activity, although its inhibitory potential is low. However, it represents a lead compound in many studies, leading to the development of a number of KDM1A-targeting derivatives [163]. These compounds inhibit the activity of KDM1A through the covalent modification of its cofactor, FAD. The addition of side groups to the phenyl ring or the N-alkylation of tranylcypromine derivatives has been shown to increase the efficacy of KDM1A inhibitors [164]. In addition to tranylcypromine, other compounds that may inhibit KDM1A include other MAO inhibitors such as pargyline, peptide- and polyamine-based inhibitors, non-peptidic propargylamines, non-peptidic compounds mimicking histone tails, benzohydrazides, phenyloxazole derivatives, amino thiazoles, thiazole sulfonamides, triazole dithiocarbamate hybrids, pyrimidine thiourea hybrids, namoline, and geranyl geranoic acid [164]. Propargylamines, which are peptide- and polyamine-based inhibitors, inhibit KDM1A as suicide inhibitory compounds through the covalent modification of FAD. Derivatives of hydrazines, such as benzohydrazides, have been found to be the most effective inhibitors of KDM1A [165]. These hybrids represent a novel class of inhibitors with anticancer properties, exhibiting considerable demethylase inhibition potential [114].

In addition to the above-mentioned KDM1A inhibitors, many potent KDM1A inhibitors with IC_50_ values in the nanomolar range (9.8–77 nM) have been found with the ability to inhibit the proliferation of MLL-rearranged leukemia cells [155]. These inhibitors exhibit EC_50_ values in the range of 10–350 nM but are non-toxic to many other tumor cells [155]. These inhibitory compounds belong to the cyclopropylamine series, and they are extremely selective for MLL-rearranged leukemia cells. Furthermore, these cyclopropylamine-based compounds do not exert toxicity, in contrast to many other KDM1A inhibitors, and hence, they may serve as useful therapeutics for MLL-rearranged leukemia cells [155]. Although KDM1A is a candidate target for treating MLL involving KDM1A, however treatment with KDM1A inhibitor alone is associated with risk of toxicity and many other side effects [157]. Recent studies have suggested combinatorial therapies, i.e., approaches involving the inhibition of DOT1L (an H3K79 methyltransferase) and the bromo-domain protein BRD4, together with the inhibition of KDM1A, to treat MLL-rearranged leukemia [166].

Moreover, as with MLL, KDM1A is a potential drug target in other subtypes of AML [167]. In the case of acute promyelocytic leukemia (APML), the use of all-trans retinoic acid (ATRA) to induce the differentiation of leukemic blasts is a standard therapy, but it is associated with remission risk [157]. ATRA alone is insufficient as a cure, and synergistic therapy with anthracycline or arsenic trioxide is required [157]. In the case of AML, single-agent treatment is rarely curative. Hence, treatment requires other options, such as the inhibition of KDM1A together with chemotherapies historically effective for APML therapy. Concomitant drug treatment (a KDM1A inhibitor together with an HDAC inhibitor) is another alternative option, as the inhibition of KDM1A aggravates the cell cycle arrest and apoptosis of breast cancer and glioblastoma cells induced by HDAC inhibitors [153, 167, 168]. The side effects of anemia and thrombocytopenia in response to this concomitant therapy can be treated by transfusions. SP2509 is a novel KDM1A antagonist, and its treatment attenuates the association of KDM1A with CoREST, along with enhancing H3K4Me3 in gene promoters and increasing p21, p27, and C/EBPα levels in cultured AML cells [158]. Moreover, treatment with this novel KDM1A antagonist inhibited the growth of AML colony cells and induced differentiation in cultured, as well as primary, AML blasts [169]. However, in contrast to MLL fusion protein treatment, SP2509 treatment triggered apoptosis in AML cells expressing mutant NPM1 [170]. Although SP2509 is an effective agent for treating AML, concomitant treatment with the pan-HDAC inhibitor panobinostat (PS) enhanced the efficiency of each agent as compared to that of each agent alone [171, 172]. Co-treatment of PS and SP2509 effectively improved the survival of mice engrafted with human AML cells without exerting any toxicity [158]. Thus, concomitant inhibitor treatment may serve as an effective and promising therapy against AML, although further investigation and preclinical trials are warranted with the aim of identifying an effective KDM1A inhibitor with improved potency and reduced side effects.

In the case of small-cell lung cancer (SCLC), a cyclopropylamine-based KDM1A inhibitor, GSK2879552, was recently discovered to serve as a mechanism-based irreversible inactivator of KDM1A [144]. The DNA hypomethylation of a signature set of probes was observed in SCLC cell lines that exhibited growth inhibition in response to GSK2879552 treatment [144]. Hence, the discovery of this small potent inhibitor of KDM1A suggests that it may serve as a predictive biomarker. Although GSK2879552 is currently under clinical development to investigate the anti-tumor potential of KDM1A inhibition in SCLC, this targeted mechanistic approach in combination with its role as a predictive biomarker makes the inhibition of KDM1A an exciting therapeutic drug target for SCLC treatment [144]. To date, three inhibitors of KDM1A are undergoing phase I clinical trials for the treatment of AML and SCLC [165].

In addition to the outlined issues, one further challenge is the targeting of the CoREST/KDM1A complex, which is involved in several functions and interacts with several protein complexes [173]. The identification of novel inhibitors with mechanisms of action other than the formation of covalent/non-covalent interactions may aid in the development of KDM1A-targeting drugs.

## Conclusions

KDM1A is a unique epigenetic modifier with the ability to maintain interactions with a variety of different protein complexes, noncoding RNAs, microRNAs, and transcription factors. The functional significance of KDM1A is maintained by its interactions at multiple sites in the genome, particularly its binding to promoters and enhancers. The complex and unique structure of KDM1A enables its binding to various other protein complexes and the inhibition or activation of gene expression. The interaction of KDM1A with different promoters, transcription factors, and protein complexes allows this protein to control the cellular oncogenic program as an important epigenetic modifier. Moreover, the involvement of KDM1A in oncogenesis and development make it an attractive therapeutic target. Detailed investigation of KDM1A as an epigenetic modifier and the mechanisms underlying its activity represents a major research challenge. In summary, the analysis of KDM1A-containing repressive and stimulatory complexes and the identification of molecular signals that affect the function of KDM1A-containing complexes are necessary for a complete understanding of epigenetic modifications and their roles in stem cell differentiation and oncogenic progression.

## Abbreviations

FAD: flavin adenine dinucleotide
KDM1A: lysine-specific histone demethylase 1A
AOL: amine oxidase-like domain
SWIRM: Swi3p/Rsc8p/Moira domain/small alpha helical domain
DNMT1: DNA methyltransferase 1
CoREST: RE1-silencing transcription factor (REST) corepressor
NuRd: Mi-/nucleosome remodeling and deacetylase
AR & ER: androgen & estrogen receptors
HDAC1: histone deacetylase 1
TAL1: T cell acute lymphocytic leukemia protein-1
EMT: epithelial–mesenchymal transition
UTR: untranslated region
TERRAs: RNAs encoded by telomeric sequences
VEGF-A: vascular endothelial growth factor A
LNCaP: lymph node carcinoma of prostate
DKK1: Dickkopf-1
LGR5: leucine-rich repeat-containing G-protein-coupled receptor 5
GSK3β: glycogen synthase kinase 3β
USP22 & 28: ubiquitin-specific peptidase 22 & 28
BMP2: bone morphogenetic protein 2
CDKN1A: cyclin-dependent kinase inhibitor 1A
GATA6: GATA binding protein 6
SIRT1: NAD-dependent deacetylase sirtuin-1
AML: acute myeloid leukemia
MLL: mixed-lineage leukemia
T-ALL: T cell acute lymphoid leukemia
PKA: protein kinase A
MAO: monoamine oxidase
SCLC: small-cell lung cancer
